# Seasonal variation in the prevalence of Gram-negative bacilli in sputum and urine specimens from outpatients and inpatients

**DOI:** 10.20407/fmj.2021-003

**Published:** 2021-08-20

**Authors:** Yusuke Kito, Kazunobu Kuwabara, Kiyotaka Ono, Kenichi Kato, Tatsuyoshi Yokoi, Kohki Horiguchi, Keisuke Kato, Masahiro Hirose, Tomomi Ohara, Kenta Goto, Yumi Nakamura, Yoshikatsu Koike, Takahiko Horiguchi

**Affiliations:** 1 Department of Respiratory Medicine II, Fujita Health University, School of Medicine, Nagoya, Aichi, Japan; 2 Department of Clinical Laboratory, Fujita Health University Bantane Hospital, Nagoya, Aichi, Japan; 3 Pharmaceutical Department, Fujita Health University Okazaki Medical Center, Okazaki, Aichi, Japan

**Keywords:** *Pseudomonas aeruginosa*, *Klebsiella pneumoniae*, Hospital infection, Seasonality

## Abstract

**Objectives:**

To determine whether the prevalence of gram-negative bacilli (GNB; *Pseudomonas aeruginosa*, *Klebsiella pneumoniae*, and *Escherichia coli*) in sputum and urine specimens from outpatients and inpatients differed by season and according to temperature and humidity changes.

**Methods:**

In this retrospective study, microbiologic data for adult patients from 2008 to 2019 were retrieved from the electronic database of a hospital in Japan. Data were categorized by specimen type (sputum and urine) and specimen collection (outpatient and inpatient). Associations between variables were assessed using Spearman’s rank correlation coefficients. Differences between groups were assessed using Pearson’s chi-square test and analysis of discrete variance.

**Results:**

Among inpatients, the frequencies of *P. aeruginosa* and *K. pneumoniae* isolation from sputum specimens were higher in summer and autumn. The frequency of *P. aeruginosa* isolation from urine specimens was higher in autumn. These seasonal trends were observed in specimens from both outpatients and inpatients. No seasonal trend was observed in the frequency of *E. coli* isolation. Mean monthly temperature was positively correlated with the frequency of isolating *P. aeruginosa* (r=0.2198, p=0.0081) and *K. pneumonia*e (r=0.3443, p=0.00002) from sputum as well as with the frequency of isolating *K. pneumoniae* (r=0.1905, p=0.0222) from urine. Mean monthly humidity was positively correlated with the frequency of isolating *K. pneumoniae* (r=0.2602, p=0.0016) from sputum.

**Conclusions:**

GNB were isolated more frequently in summer and autumn than in other seasons. These seasonal trends were observed for both outpatient and inpatient specimens. Seasonality should be considered for optimal infection control of GNB in hospitals.

## Introduction

Hospital-acquired infections are a global problem. It has been estimated that 3.2% of hospital inpatients receiving acute care will develop a hospital-acquired infection.^1^ Pneumonia and urinary tract infections are the most common nosocomial infections. The causative agents are most often gram-negative bacilli (GNB) such as *Pseudomonas aeruginosa*, *Klebsiella pneumoniae*, and *Escherichia coli*.^2–5^

Environmental temperature and humidity are important determinants of pathogen survival. The incidence of infectious diseases can exhibit seasonal variations. These patterns are specific to individual pathogens and their hosts, modes of transmission, and environmental characteristics.^6,7^ GNB infections are more common during seasons with high temperature and humidity.^7–9^ However, some reports have indicated no changes in the frequency of GNB isolation according to climate.^10^ Thus, studies of seasonal effects on the frequency of microbial isolation may depend on the climate of the region under study.

The environmental conditions within a hospital are generally regulated by air conditioning, with the temperature and humidity kept constant. Therefore, the impact of temperature and humidity on the frequency of isolating pathogenic microorganisms may differ between outpatients and inpatients. However, to our knowledge, no studies have compared the seasonality of microbial isolation frequency between inpatient and outpatient specimens. Understanding differences in the impact of seasonality on microbial isolation frequency between outpatients and inpatients will help in formulating infection control protocols.

The purpose of this study was to determine whether the frequency of isolating GNB (*P. aeruginosa*, *K. pneumoniae*, and *E. coli*) from sputum and urine specimens differed by season (according to temperature and humidity) in both outpatients and inpatients.

## Methods

### Specimens and microbiologic data

We retrieved microbiologic data for adult patients retrospectively from the electronic database of Fujita Health University Second Education Hospital (latitude 35.144, longitude 136.891). The hospital is a 370-bed secondary medical institution in Nagoya, Japan, with 10 intensive care beds. Data for all specimens collected from January 2008 to December 2019 for microbiological testing were extracted, regardless of the reason for collection. All specimens collected for performing clinical tests were included. Specimens obtained from patients younger than 16 years of age and specimens not intended for clinical testing were excluded.

Data were categorized by specimen type (sputum and urine) and specimen collection (outpatient and inpatient) by order of entry. We retrieved culture-positive microbiologic data for *P. aeruginosa*, *K. pneumoniae*, and *E. coli*.

The isolation frequency for each microorganism was calculated by dividing the number of isolates by the total number of specimens per month. We defined March, April, and May as spring; June, July, and August as summer; September, October, and November as autumn; and December, January, and February as winter. Isolation frequency was calculated for each season.

The average monthly temperature (°C) and humidity (%) for each of the 144 months of the study were obtained from the Japan Meteorological Agency. We assessed the relationships between seasonal changes in temperature and humidity and the isolation frequency for each microorganism for each month.

This study was approved by the Medical Research Ethics Committee of Fujita Health University (approval no. HM20-120). The study conformed to the principles outlined in the Declaration of Helsinki. The need for informed consent was waived because of the retrospective nature of the study.

### Statistical analysis

Continuous data were expressed as means. Categorical data were expressed as counts and percentages. Associations between environmental variables (temperature and humidity) and isolation frequency were assessed using Spearman’s correlation coefficients. Differences between groups were assessed using Pearson’s chi-square tests and analysis of discrete variance, as appropriate. Values of p<0.05 were considered statistically significant. StatMate version 3.19 (ATMS Co., Ltd., Tokyo, Japan) was used for all statistical analyses.

## Results

During the 12-year (144-month) study period, 8746 sputum specimens and 9627 urine specimens were included in the analysis (Table 1). The percentages of sputum specimens collected during the spring, summer, autumn, and winter were 25%, 23%, 25%, and 26%, respectively. The percentages of urine specimens collected during the spring, summer, autumn, and winter were 24%, 27%, 26%, and 23%, respectively. The frequencies of isolation of microorganisms from sputum and urine were 12.1% and 4.3% (*P. aeruginosa*), 11.5% and 5.4% (*K. pneumoniae*), and 6.4% and 29.0% (*E. coli*).

**Figure 1:**
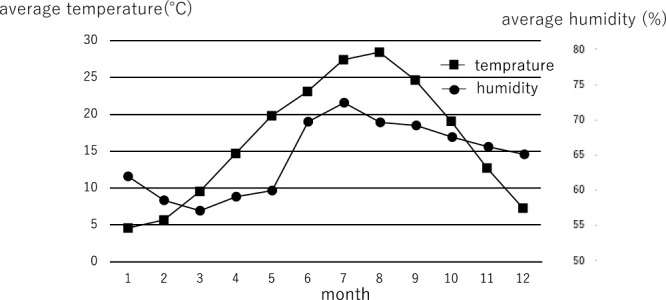
Monthly average temperature and humidity from 2008 to 2019 in Nagoya, Japan. Data were obtained from the Japan Meteorological Agency.

Based on climate data obtained from the Japan Meteorological Agency database, the average temperature and humidity for each month and each season over the 12-year study period were determined (Figure 1 and Table 2).

### Microorganism isolation frequency by season

**Figure 2:**
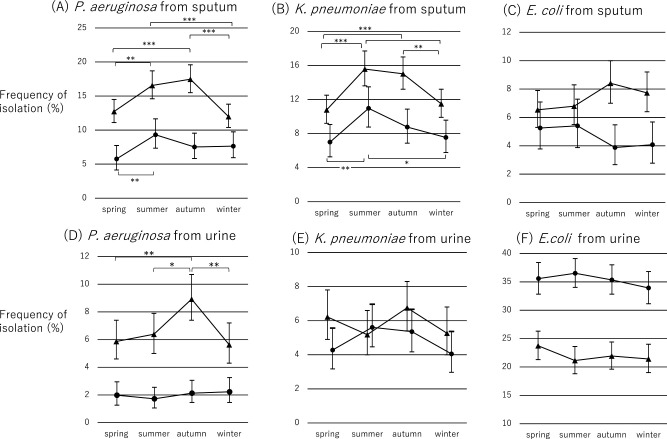
Differences in the frequency of isolating Gram-negative bacilli by season (spring, summer, autumn, winter). Seasonal differences are shown in the isolation frequency of (A) *Pseudomonas aeruginosa* from sputum specimens; (B) *Klebsiella pneumoniae* from sputum specimens; (C) *Escherichia coli* from sputum specimens; (D) *P. aeruginosa* from urine specimens; (E) *K. pneumoniae* from urine specimens; and (F) *E. coli* from urine specimens. ● Outpatient, ▲ inpatient. * p<0.05, ** p<0.01, *** p<0.001.

We examined the frequency of isolation of each microorganism by season (Figure 2). The frequency of *P. aeruginosa* isolation in sputum specimens from outpatients was significantly higher in summer than in spring (p<0.01). The frequencies of *P. aeruginosa* and *K. pneumoniae* isolation in sputum specimens from inpatients were significantly higher in summer and autumn compared with winter and spring. The frequency of *P. aeruginosa* isolation in urine specimens from inpatients was higher during autumn than during other seasons. The frequency of *E. coli* isolation did not differ significantly by specimen type or season for both inpatients and outpatients.

### Associations of microorganism isolation frequency with temperature and humidity

**Figure 3:**
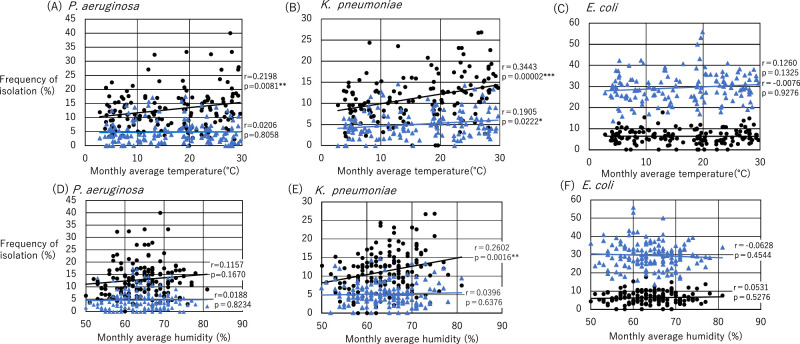
Correlations of microorganism isolation frequency from sputum and urine with temperature and humidity for inpatients and outpatients. (A) Correlation between *Pseudomonas aeruginosa* isolation frequency and monthly average temperature. (B) Correlation between *Klebsiella pneumoniae* isolation frequency and monthly average temperature. (C) Correlation between *Escherichia coli* isolation frequency and monthly average temperature. (D) Correlation between *P. aeruginosa* isolation frequency and monthly average humidity. (E) Correlation between *K. pneumoniae* isolation frequency and monthly average humidity. (D) Correlation between *E. coli* isolation frequency and monthly average humidity. r: Spearman’s rank correlation coefficient. ● Sputum specimen, ▲ urine specimen. * p<0.05, ** p<0.01, *** p<0.001.

The relationships between the isolation frequency of each microorganism and average monthly temperature and humidity are shown in Table 3. The frequency of *P. aeruginosa* isolation from sputum specimens correlated positively with mean monthly temperature (r=0.2198, p=0.0081); however, no such correlation was observed for urine specimens. The frequencies of *P. aeruginosa* isolation from sputum and urine were not correlated with mean monthly humidity. We also examined the relationships between climate and isolation frequency among inpatients and outpatients (Table 3, Figure 3). Among inpatients, the frequency of *P. aeruginosa* isolation from sputum specimens correlated with monthly average temperature (r=0.1929, p=0.0205), whereas no such correlation was observed for outpatients.

The frequencies of *K. pneumoniae* isolation from sputum and urine samples were positively correlated with monthly mean temperature (r=0.3443, p=0.00002 and r=0.1905, p=0.0222, respectively). The frequency of *K. pneumoniae* isolation from sputum was positively correlated with monthly mean humidity (r=0.2602, p=0.0016). The frequency of *K. pneumoniae* isolation from sputum was positively correlated with monthly mean temperature among both outpatients (r=0.2744, p=0.0087) and inpatients (r=0.2830, p=0.0006). Among inpatients, the frequency of *K. pneumoniae* isolation from sputum was positively correlated with monthly mean humidity (r=0.2507, p=0.0024). Among outpatients, the frequency of *K. pneumoniae* isolation from urine specimens was positively correlated with monthly mean temperature (r=0.2425, p=0.0034).

The frequency of *E. coli* isolation was not correlated with monthly mean temperature or humidity in either specimen type for outpatients or inpatients.

## Discussion

In this study, we investigated associations between the frequencies of isolating GNB from sputum and urine specimens and climatic factors. *P. aeruginosa* and *K. pneumoniae* were more frequently isolated from sputum specimens during the summer and autumn, while *P. aeruginosa* was more frequently isolated from urine specimens during the autumn. Our study showed that *P. aeruginosa* and *K. pneumoniae* were isolated more frequently in warm and humid climates. The effects of climatic variations was observed for both outpatient and inpatient specimens. The frequency of *E. coli* isolation was not affected by season or climate.

In hospitals, temperature and humidity are thought to be controlled by air conditioning equipment. However, inpatients may have different clinical characteristics than outpatients, such as reduced activities of daily living, hyperthermia, dysphagia, and use of urinary catheters. In addition, there is a risk of microorganisms being introduced to inpatients by outsiders and contact infection through healthcare workers. These factors may have affected the seasonality of GNB isolation observed among inpatients in this study. Additional studies are needed to examine these hypotheses.

Several studies have reported that the incidence of GNB infections peaks in warmer months.^9,11^ *P. aeruginosa* is frequently isolated from hospitalized patients and is an important target for nosocomial infection control. *P. aeruginosa* grows well at a temperature of 37°C but can survive at a wide range of temperatures (from 4°C to 42°C).^12^ As reported in a previous study, the incidence of *P. aeruginosa* infection increases by 17% for each 5.6°C increase in the external temperature.^9^ In our study, the frequency of *P. aeruginosa* isolation from sputum correlated with temperature only for inpatient specimens. A study in Brazil identified correlations between *P. aeruginosa* urinary tract infection and temperature and between hospital-associated pneumonia and humidity.^11^ However, in the present study, no correlation was observed between the frequency of *P. aeruginosa* isolation in urine samples and humidity. The region of Brazil where the study was conducted was more humid than the area surrounding our hospital, hence the discrepant findings between the two studies may relate to differences in environmental factors.

*K. pneumoniae* is the most heat-tolerant of all enteric pathogens^13^ and can survive at higher temperature than other enteric pathogens.^14^ *K. pneumoniae* can colonize mucosal surfaces and then spread from the mucosa to other tissues,^15^ causing pneumonia, urinary tract infections, and bloodstream infections. This organism causes 10% of nosocomial pneumonias and 6%–17% of nosocomial urinary tract infections.^4,16^ In our study, we observed a seasonal impact on the frequency of *K. pneumoniae* isolation from sputum for both outpatients and inpatients. Although there have been no reports examining seasonal variation in the frequency of *Klebsiella* isolation from sputum and urine specimens, a previous study of nosocomial bloodstream infection reported a higher incidence of *Klebsiella* in warm climates.^17^ The observed seasonality in bloodstream *Klebsiella* infections may result from seasonality in primary urinary tract infections and pneumonias.^7^

*E. coli* is an important bacterium causing nosocomial infections and is an increasingly drug-resistant microorganism.^3^ Our results showed no seasonal variation in the frequency of *E. coli* isolation from urine and sputum. This result is in contrast with those of international studies showing seasonality in the frequency of *E. coli* isolation.^8,18^ In the present study, the frequency of *E. coli* isolation in both sputum and urine samples was not affected by climate. This contrasts with the seasonality of *P. aeruginosa* and *K. pneumoniae* isolation. Interestingly, a report from England on patients with *E. coli* bacteremia concluded that the frequency of *E. coli* isolation varied widely within the same country and was poorly correlated with climate.^10^ In our hospital, we observed no seasonality in the frequency of *E. coli* isolation. Therefore, the seasonality of *E. coli* isolation may be more variable than that of *P. aeruginosa* and *K. pneumoniae* isolates, and may depend on the surveyed region.

The 2019 guidelines for community acquired pneumonia by the American Thoracic Society consider local factors in assessing risk of infection by resistant bacteria such as *P. aeruginosa*, although specific methods for evaluation have not been described.^19^ Based on historical data, an increase in infections by GNB may occur during the summer.^8–11^ Our data likewise showed that GNB were isolated at higher frequencies in the summer as well as the autumn. Seasonal variation in isolation frequency was observed for both outpatients and inpatients. Our data indicate that seasonality is a factor that should be considered in nosocomial infection control. Japan’s climate has higher humidity during the warmer months, in contrast to western climates with lower humidity during the warmer months. Seasonal differences in temperature and humidity may have affected the results of individual studies. Discrepancies between findings indicate the need for regional epidemiologic studies. In addition, because of global warming, the risk of transmission of GNB is expected to increase over time. Hence, there is a need to assess changes in the frequency of isolating GNB from sputum and urine specimens over time associated with climatic variations.

### Limitations

Several limitations of this study should be acknowledged. First, the study was conducted at a single institution, and different results may be obtained in other regions or types of institutions (i.e., cancer centers). Second, we did not investigate patients’ clinical backgrounds, including underlying disease or the factors that led to culture testing. Finally, because inpatient and outpatient specimens were identified by order of entry, it is possible that the data for outpatient-setting microorganisms may have been misclassified as inpatient-setting.

## Conclusion

Among inpatients, the frequencies of *P. aeruginosa* and *K. pneumoniae* isolation from sputum specimens were higher during summer and autumn than during winter and spring, while the frequency of *P. aeruginosa* isolation from urine samples was higher in autumn. Similar trends were observed for outpatient specimens. In contrast, there was no seasonal variation observed in the frequency of *E. coli* isolation. Frequency of *E. coli* isolation was not correlated with monthly mean temperature or humidity for either specimen type. These results indicate that seasonality and geography need to be considered in the design of GNB infection control protocols.

## Figures and Tables

**Table1:** Patient background characteristics and microbiologic specimen data

Total, n=18373		Sputum specimen (n=8746)	Urine specimen (n=9627)
age, mean±SD		75.5±14.1	73.2±15.8
male, n (%)		5226 (60)	4580 (48)
Inpatient,n (%)		3100 (35)	5042 (52)
Number of samples by season			
spring, n (%)		2223 (25)	2309 (24)
summer, n (%)		2047 (23)	2578 (27)
autumn, n (%)		2210 (25)	2535 (26)
winter, n (%)		2266 (26)	2205 (23)
Isolation frequency			
*P. aeruginosa*	total, n (%)	1062 (12.1)	412 (4.3)
	outpatient, n (%)	236 (7.6)	103 (2.0)
	inpatient, n (%)	826 (14.6)	309 (6.7)
*K. pneumoniae*	total, n (%)	1007 (11.5)	517 (5.4)
	outpatient, n (%)	264 (8.5)	248 (4.9)
	inpatient, n (%)	743 (13.2)	269 (5.9)
*E. coli*	total, n (%)	560 (6.4)	2796 (29.0)
	outpatient, n (%)	144 (4.6)	1785 (35.0)
	inpatient, n (%)	416 (7.4)	1011 (22.1)

*P. aeruginosa*, *Pseudomonas aeruginosa*; *K. pneumoniae*, *Klebsiella pneumoniae*; *E. coli*, *Escherichia coli.* March, April, and May were defined as spring; June, July, and August as summer; September, October, and November as autumn; and December, January, and February as winter. SD, standard deviation.

**Table2:** Average temperature and humidity in Nagoya, Japan, during each season (2008–2019)

	average temperature (°C)	average humidity (%)
All period, mean±SD	16.4±0.4	64.2±2.1
Spring, mean±SD	14.7±0.8	58.5±3.1
Summer, mean±SD	26.3±0.6	69.8±3.3
Autumn, mean±SD	18.8±0.6	67.0±2.4
Winter, mean±SD	5.8±0.8	61.5±2.8

The average monthly temperature and humidity data for each season in Nagoya, Japan, were obtained from the Japan Meteorological Agency. March, April, and May were defined as spring; June, July, and August as summer; September, October, and November as autumn; and December, January, and February as winter. SD, standard deviation.

**Table3:** Correlations between the frequency of isolation of each microorganism and average monthly temperature and humidity

				temperature		humidity	
	specimen	location	Isolation, n (%)	r	p-value	r	p-value
*P. aeruginosa*	Sputum	total	1062 (12.1)	0.2198	0.0081**	0.1157	0.1670
		outpatient	236 (7.6)	0.1093	0.1093	0.1808	0.0530
		inpatient	826 (14.6)	0.1929	0.0205*	0.0826	0.3248
	Urine	total	412 (4.3)	0.0206	0.8058	0.0188	0.8234
		outpatient	103 (2.0)	–0.022	0.7879	–0.0245	0.7705
		inpatient	309 (6.7)	0.0499	0.5521	0.0820	0.3284
*K. pneumoniae*	Sputum	total	1007 (11.5)	0.3443	0.00002***	0.2601	0.0016**
		outpatient	264 (8.5)	0.2744	0.0087**	0.1016	0.22539
		inpatient	743 (13.2)	0.2830	0.0006***	0.2507	0.0024*
	Urine	total	517 (5.4)	0.1905	0.0222*	0.0396	0.6376
		outpatient	248 (4.9)	0.2425	0.0034**	0.0945	0.2597
		inpatient	269 (5.9)	0.0653	0.4370	–0.0065	0.9381
*E. coli*	Sputum	total	560 (6.4)	–0.0076	0.9276	0.0531	0.5276
		outpatient	144 (4.6)	0.075	0.3724	0.0143	0.8647
		inpatient	416 (7.4)	–0.0525	0.5322	0.0414	0.6225
	Urine	total	2796 (29.0)	0.1260	0.1325	–0.0628	0.4544
		outpatient	1785 (35.0)	0.0882	0.2933	–0.0635	0.4496
		inpatient	1011 (22.1)	–0.0034	0.9678	–0.0970	0.2474

*P. aeruginosa*, *Pseudomonas aeruginosa*; *K. pneumoniae*, *Klebsiella pneumoniae*; *E. coli*, *Escherichia coli.* r: Spearman’s rank correlation coefficient. * p<0.05, ** p<0.01, *** p<0.001.
